# Critical role of climate change in plant selection and millet domestication in North China

**DOI:** 10.1038/s41598-018-26218-6

**Published:** 2018-05-18

**Authors:** Xiaoyan Yang, Wenxiang Wu, Linda Perry, Zhikun Ma, Ofer Bar-Yosef, David J. Cohen, Hongbo Zheng, Quansheng Ge

**Affiliations:** 1 Key Lab. of Land Surface Pattern and Simulation, Institute of Geographical Sciences and Natural Resources Research, Chinese Academy of Sciences, Beijing, 100101 China; 2 The Foundation for Archaeobotanical Research in Microfossils, Alexandria, VA USA; 3 Department of Anthropology, George Washington University, Washington, DC USA; 4 School of Cultural Heritage, Northwest University, Xi’an, 710069 China; 5 Department of Anthropology, Harvard University, Cambridge, 02138 MA USA; 6 Department of Anthropology, National Taiwan University, Taipei, 10617 Taiwan; 7 Research Center for Earth System Science, Yunnan University, Kunming, 650091 China

## Abstract

While North China is one of the earliest independent centers for cereal domestication in the world, the earliest stages of the long process of agricultural origins remain unclear. While only millets were eventually domesticated in early sedentary societies there, recent archaeobotanical evidence reported here indicates that grasses from the Paniceae (including millets) and Triticeae tribes were exploited together by foraging groups from the Last Glacial Maximum to the mid-Holocene. Here we explore how and why millets were selected for domestication while Triticeae were abandoned. We document the different exploitation and cultivation trajectories of the two tribes employing ancient starch data derived from nine archaeological sites dating from 25,000 to 5500 cal BP (LGM through mid-Holocene) in North China. With this diachronic overview, we can place the trajectories into the context of paleoclimatic reconstructions for this period. Entering the Holocene, climatic changes increased the yield stability, abundance, and availability of the wild progenitors of millets, with growing conditions increasingly favoring millets while becoming more unfavorable for grasses of the Triticeae tribe. We thus hypothesize that climate change played a critical role in the selection of millet species for domestication in North China, with early domestication evidenced by 8700 cal BP.

## Introduction

Unraveling the mechanisms behind the selection of particular plants for cultivation and domestication is key to understanding the origins of dry-land farming in North China, one of the earliest independent domestication centers in the world^1^. While pioneering research focused on the domesticated millets, recent research on archaeological plant remains indicates more complex patterns of foraging before domestication, with other grasses, in addition to wild millet, likely exploited as food sources^2–9^. Research must now be directed toward the human choices that led to the favoring of wild millet’s cultivation and domestication as a food source.

**Figure 1:**
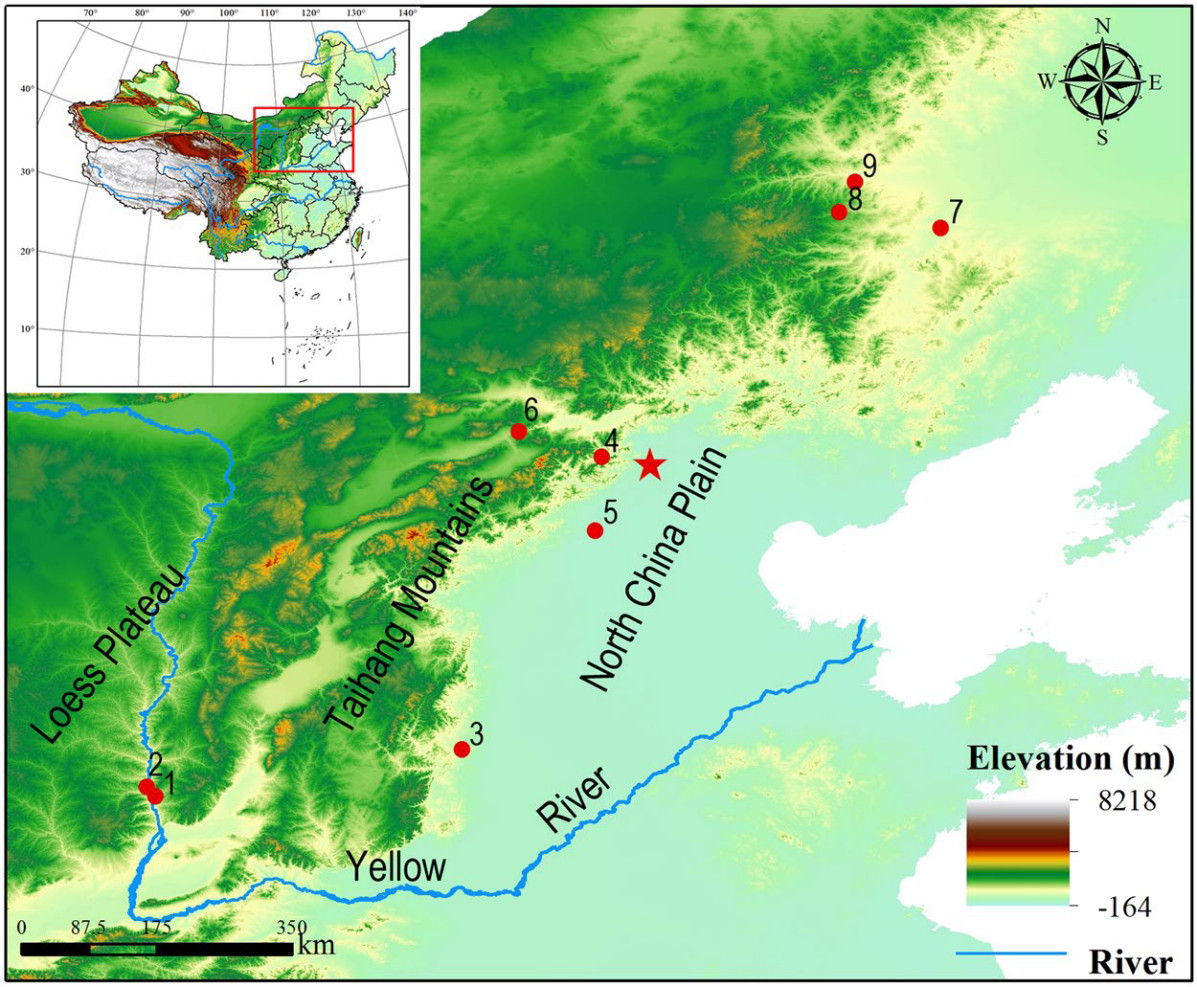
The study region in North China showing the nine archaeological sites with starch remains analyzed. Red star indicates Beijing. 1. Shizitan; 2. Longwangchan; 3. Cishan; 4. Donghulin; 5. Nanzhuangtou; 6. Jiangjialiang; 7. Sanjianfang; 8. Luojiayingzi; 9. Jian’gou. Maps generated using DIVA-GIS 7.5 (http://www.diva-gis.org/).

Archaeobotanical macro-remains in Late Paleolithic hunter-gatherer sites in North China are often poorly preserved or not clearly associated with food-production activities, meaning they cannot be directly linked to objects used to cook or eat the plants, and thus to human consumption. This study is based on ancient starch grains identified on grinding stones used directly in food production from nine sites dating from the Late Pleistocene Last Glacial Maximum through the Holocene Climatic Optimum, *ca*. 25,000 to 5500 cal BP (calibrated years before present, based on radiocarbon dates) (Fig. 1). Because these remains of edible plants are found on the grinding stones, we can reliably assume humans were consuming them. While previous starch studies focused on remains from individual sites^2–9^, these data from multiple sites afford us a long-term, diachronic view for uncovering the changing relationships between people and the cereals they consumed during the cultivation and domestication processes. Exclusively studying starches on such tools also allows us to reliably assume that we are making consideration only of residues from plants whose seeds were ground for consumption. This study demonstrates that foragers who became initial cultivators ate the seeds of grasses not only from the Paniceae tribe, to which millets—the initial cereal domesticates—belong, but also from Triticeae. We assume that due to their recovery from the surfaces of food-processing tools, the changing relative percentages of these tribes’ starch granules are a reliable indicator of cereal food preferences. Looking at the relative percentages between starches from the different tribes provides insights to the trajectory that led to millet as the preferred cereal for domestication. We next place these plant data into the context of climatic fluctuations, which shows correlations, and likely causation, between environmental change and changes in the cereal plants’ distribution and exploitation patterns. We hypothesize that climate change played a crucial role in causing cultivating foragers to increasingly abandon Triticeae species and select Paniceae members as the most suitable plants for domestication, and we model why this happens. We also recognize the limits of starch identifications, such as being limited to the tribe level, and call for additional evidence to test this hypothesis.

The origins of agriculture are best understood today in southwestern Asia, where broad spectrum exploitation of edible plants was followed by the selection of a limited number of species for cultivation, with resultant domestication emerging during the early Neolithic period^10–14^. The cereal domesticates were barley (*Hordeum vulgare*) and wheat (*Triticum* spp.), members of the tribe Triticeae^11,15^. For North China, the more limited archaeological evidence can be analyzed in reference to southwestern Asia models^1^. In order to model the process leading to the farming of domesticated millet, we treat the starch evidence from the nine archaeological sites as representative of distinct stages that led to eventual domestication. Our evidence shows the differing consumption of grasses in both the Triticeae and Paniceae tribes and reflects changing patterns in human-plant relationships^5,6,8^. The stages in this process include collecting wild cereals by mobile foraging groups; early cultivation of wild cereals possibly involving management and selection; further cultivation by less mobile/sedentary bands; cultivation of wild millets with planting and harvesting near permanent Neolithic villages; and the agricultural production of domesticated millet. In particular, we document the decreasing consumption of members of the Triticeae grasses and increase in Paniceae members—likely the wild progenitors of foxtail millet (*Setaria italica*) and broomcorn millet (*Panicum miliaceum*)—as they come to be cultivated, farmed, and domesticated. Other terminology used for such stages in this evolutionary continuum, after Harris^16^, include “pre-domestication cultivation”, “semi-domestication”, and a later “full domestication”^17^. Although starch granules cannot be identified to the species, we can infer the wild or domesticated millet species are represented at the various sites based on identifications of rare finds of charred macro-remains of these millets^9^. The starch evidence raises a critical question: why did the exploitation of Triticeae and Paniceae grasses in North China ultimately result in the selection of millets as the crop to be domesticated for a dry-land farming system while the grasses of the Triticeae tribe were abandoned?

Documenting the process of millet domestication requires evidence for cultivation of the wild species within a region where wild cultivation and dry-land farming could be successful in producing the surplus needed to feed growing semi-sedentary to sedentary populations. The region had to feature the required growing conditions for millets. Foxtail millet has a growing season of 60–120 days while broomcorn millet’s is 45–100 days; each has certain heat requirements, such as cardinal temperatures for germination for broomcorn millet of 30–40 °C and for foxtail millet of 20–30 °C, but also, these cereals are relatively unable to withstand frost (compared to wheat or barley)^18^. Researchers in East Asia, following models from southwestern Asia, have argued that shifting climatic conditions played an important role in the selection of cereals for domestication^1,15^. Finer resolution, local environmental proxies from pollen, loess, and speleothem records^19^ can provide reconstructions of climate fluctuations and environmental changes from the Late Pleistocene to the Holocene Optimum in which to place our data, allowing us to hypothesize possible mechanisms responsible for the divergent paths of the grass tribes, with millets becoming more intensively cultivated and the Triticeae grasses being abandoned.

## Results

The oldest starch remains were observed on the earliest grinding slabs excavated in China, at the earliest-known microblade industry sites, at Longwangchan (Shaanxi) and Shizitan (Shanxi), located on the Loess Plateau along the middle Yellow River valley (Fig. 1). The economies of these Late Paleolithic sites feature the collecting of wild plants and hunting by mobile foraging groups^20^. We collected seven residue samples from the surface of a grinding slab recovered from Layer 5 (~26/25,000 cal BP) at Locality 1 of Longwangchan (36°09′45″N, 110°26′15″E)^21^ and recovered 60 starch granules. Of them, 46 starch granules, or 76.7% of the total assemblage (and seen in all samples), are identified as derived from grasses of the tribe Paniceae, and 9 starch granules (15%) of the tribe Triticeae (recovered from five samples) (Table S1).

Starch data from Shizitan Locality 14 (36°02′11″N, 110°32′40″E) are obtained from six samples from three grinding slabs dating 23,000–19,500 cal BP, from Layer 4 (GS2, GS3), and Layer 3 (GS1)^6^. These samples include 20 starch granules (15% of the total starch counts) identified as derived from the Paniceae, and 45 starch granules (45% of the total starch), as Triticeae (Table S2). A later locality, Shizitan 9 (13,800–11,600 cal BP), produced both tribes’ starches, as well as phytoliths and seeds of wild foxtail millet^9^. The identification of *Glumus* fungus is likely indicative of tillage at Shizitan 9, so foragers may have been cultivating wild millet^9,22^. An analogous case is the growing of wild cereals at Ohalo II in southwestern Asia^14^.

Following the Younger Dryas, new forms of sites appear in North China, represented by Nanzhuangtou (Hebei) and Donghulin (Beijing). Such sites, likely featuring more extensive wild cereal cultivation, could belong to less mobile or semi-sedentary groups. Nanzhuangtou (39°6′40″N, 115°39′25″E; 11,500–11,200 cal BP)^5,23^ features flake tools (without microblades), polished axes, more querns and mullers, clearly defined activity areas, pottery, domesticated dogs, and wild pig and chicken. At Donghulin (39°58′48″N, 115°43′36″E) (11,150 to 9500 cal BP)^2,23^, microblades are still present, but appear along with pit burials, pit-houses, and hearth pits.

In re-examining previously published data^8^, more than 1,000 starch granules were recovered from 18 samples collected at Nanzhuangtou and Donghulin. Paniceae dominate the starch assemblages (comprising more than 50% of the total), and are likely of the genera *Setaria* and *Panicum*, as each has been documented as undergoing cultivation at both sites^2,5^. Starches from Paniceae and Triticeae occur in all samples collected at Nanzhuangtou and those from the early occupational phase of Donghulin (Tables S3 and S4).

Sedentism marks a major turning point in the domestication process, as it gives rise to the earliest permanent villages and ceremonial centers and new requirements for local food production. The settlements feature planting and storage of wild millets and the earliest morphologically domesticated plants. This phase is represented by the North China Plain sites of Cishan (36°34′31″N, 114°06′43″E) and Jiangjialiang (40°11′38″N, 117°17′53″E). Cishan is an eight hectare site, culturally related to a number of village sites of the Cishan-Peiligang-Houli culture sphere on the lowland plains and dry grasslands of the middle and lower Yellow River catchment, perhaps dating ~8000–7500 cal BP^23^, while Jiangjialiang dates ~7700–5700 cal BP^24^. Excavations at Cishan uncovered the remains of temporary rounded-oval huts with grinding stones numbering more than those in domestic contexts in contemporaneous settlements. 474 storage pits (of depths up to 5 m) contained large quantities of millet remains. All the finds demonstrate the ceremonial nature of this locality. The estimated 50,000 kg of stored millet shows that systematic farming was probably well established by this time^25^, with primarily *Panicum miliaceum* identified, while *Setaria italica* appears in late contexts in small quantities^26,27^. *Panicum miliaceum* is known to inhabit an arid ecological niche, while *Setaria* grasses tend to inhabit warm temperate, subtropical, and tropical areas, leading Bestel *et al*.^28^ to posit that *Setaria* grasses would not have been a weed of broomcorn millet. The 24 residue samples taken from three querns and seven mullers uncovered in the site yielded 10 starch grains (6.7%) from the Triticeae alongside 116 starch grains (77.9%) from (likely) millets.

The Jiangjialiang excavations revealed nine square, semi-subterranean houses, marking it as a later settlement site than Cishan-Peiligang related sites with round houses. From 21 residue samples collected from three querns and two mullers found in a house, we recovered 13 starch grains from Triticeae alongside 104 starch grains from Paniceae (likely millets). The Paniceae starches make up 81.3% of the entire assemblage, and they are found in all samples at both sites, indicating widespread consumption. In contrast, the percentage of starches from the Triticeae is 10.2%. Triticeae are only found in 20–30% of the samples from both sites (Tables S5 and S6). The more limited domestic preparation of Triticeae for food during this period, including in ceremonial contexts, needs to be considered further in reconstructions of early Neolithic consumption patterns and ritual.

The final three sites date 6500–5500 cal BP and belong to the Hongshan Culture in Northeast China, known for its jade production, ceremonial sites, and more complex social organization than Cishan. The sites—Jian’gou (43°10′33.2″N, 118°40′55.7″E), Luojiayingzi (42°49′42.9″N,118°30′10.0″E), and Sanjianfang (42°38′12.7″N, 119°41′03.5″E)—are generally located within a similar environment to the other sites, with millet cultivated here since the Xinglongwa Culture, ca. 8300 cal BP, with more domesticated millet macro-remains appearing by 7650 cal BP^23,25^. We examined 12 residue samples collected from three slabs and three mullers. 290 starch granules of millets were identified in these samples making up 97% of the starch assemblage, but only four granules from Triticeae, 1.3% of the total, were recovered, from 3 samples (Table S7).

**Figure 2:**
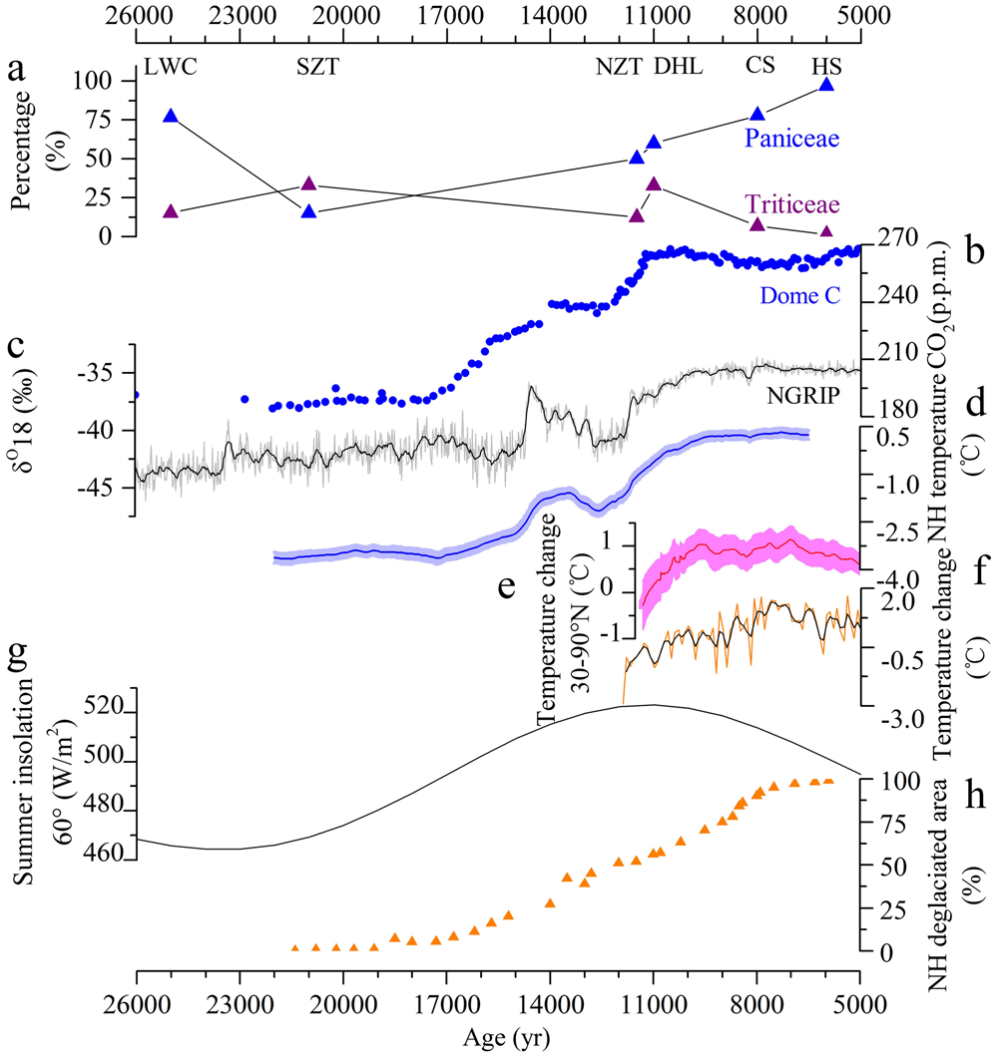
Changes of percentage of starches from LLGP to the Holocene and climate change. (**a**) Percentage of starches from LLGP to the mid-Holocene; (**b**) Atmospheric CO_2_ concentration from Dome Concordia, Antarctica^34^; (**c**) Oxygen stable isotope ratios (δ^18^O) at 20-year resolution from Greenland ice core, NGRIP^46^; (**d**) Northern hemisphere temperature change^47^; (**e**) The temperature anomaly for 30–90°N^48^; (**f**) Temperature change for China^49^; (**g**) Summer insolation at 60°N^57^. LWC: Longwangchan; SZT: Shizitan 14; NZT: Nanzhuangtou; DHL: Donghulin; CS: Cishan; HS: Hongshan culture sites, combined.

During the early Holocene, as Paniceae becomes the preferred species for cultivation, Triticeae cereals gradually fall out of favor. The Hongshan site starch data document the final decrease in the relative percentage of Triticeae by the mid-Holocene (Fig. 2a). Preserved macro-remains from mid-Holocene sites show agricultural production featuring greater reliance on domesticated broomcorn and foxtail millets. The decrease in Triticeae, shown clearly by starches, occurs as the millet agricultural systems develop associated with the rise of permanent settlements, social ranking, and more developed ritual complexes that are emerging out of increasing Neolithic cultural elaboration that begins with the Peiligang-Cishan-Houli and Xinglongwa cultures^23,25,29,30^.

## Discussion and Conclusions

While previous starch studies in North China have focused on identifications at single sites, this study considers diachronic change in starch remains through the 20,000-year time period from the LGM to the Holocene Climatic Optimum. Our focus on cereals provides evidence of inter-related trajectories in the exploitation of Paniceae and Triticeae tribe members across five stages of human-plant interactions, from collecting wild cereals by mobile foragers to agricultural production.

For Late Pleistocene sites, because macro-remains are poorly preserved, starch analysis often provides our only entryway to reconstructing plant exploitation patterns, in spite of small sample sizes and limited capability of only identifying to the tribe level. The small sample sizes, which are due to the ephemeral nature of site occupations and the complex issues of preservation and recovery, unfortunately, do not allow validation by statistical tests. The advantages of starch analysis, however, include the identification of plant use in the absence of macro-remains and the unique capability of enabling the identification of plants that were processed by specific tools, such as by grinding tools likely used in food processing, and thus can provide direct evidence of food consumption^3^. While preserved plant macro-remains are rare in earlier hunter-gatherer sites, remains from Neolithic settlements provide species-level identifications using wild vs. domesticated morphologies of millets; they also allow the recognition of broader spectra of exploited plant food resources at these sites than previously understood, including cereals, fruits, tubers, and especially nuts. Macro-remains are practically unknown for Late Pleistocene sites^9^, but based on Nanzhuangtou, Donghulin, and other sites, we would predict humans practicing both strategies of broad spectrum plant food consumption and of resource intensification.

We posit that changes in the trajectories for the two exploited tribes, featuring the increasing preference for Paniceae (likely wild millets) leading to domestication for agricultural production with concomitant decreases in Triticeae consumption, are the result of a series of choices through time by Late Paleolithic and early Neolithic groups. These were in response to the increasing availability and productive yields of certain grasses that were resultant from changing global climate and local environments. Some changes in plant physiology and distribution were also the result of human agency, likely featuring selection and tending, management, and eventual cultivation. While detailed understanding of the dynamics of human-plant interactions must await further archaeological finds and studies, here we can begin by modeling the choices made in selecting millets over Triticeae, which we hypothesize were mostly influenced by changing environmental conditions in North China.

This environmental impact hypothesis for the selection of millets and their preference over Triticeae is supported by the known effects of climate on inherent physiological traits of the two tribes of grasses^31^: these impact seasonal availability, abundance, and predictability of the plants, and observant foragers and early cultivators would have recognized such changes and perhaps promoted some through selection and management. To evaluate the role of climatic conditions in North China, we review the paleoclimatic sequence as known from recent proxies, including ice core, pollen, and speleothem profiles, and then place the archaeologically-known starch trajectories into their paleoenvironmental contexts.

During the Last Glacial Maximum (now correlated with Greenland Stadial 3, dating 27,540–23,340 cal BP)^32,33^, atmospheric CO_2_ concentrations were one-third lower (c. 180–235 ppmv) than early post-glacial levels (12,000–10,000 cal BP) (c. 265–270 ppmv)^34^ (Fig. 2b). With the strengthened winter monsoon^35^, LGM mean temperature was approximately 7–10 °C lower than today’s average, and annual precipitation was 200 to 300 mm less than at present in the study area^36^, with vegetation and permafrost zones shifted southward from current boundaries^37,38^. The combined impact of these variables on water resources and biomass productivity could have led to severely decreased food resources for hunter-gatherers in North China, who would be forced to adopt various measures to minimize risks. While higher mobility is sometimes seen as a possible solution, environmental limitations in North China during this extreme period would have made high mobility impossible, and they forced hunter-gatherers to aggregate and compete for resources that had become more patchy^19,39^. During LGM, Paleolithic sites disappear from north of 41°N, likely with migration southward into North China^39^.

During the Terminal Pleistocene, with amelioration, the global trend toward increasing population may apply in North China, as well^40,41^. Diversification of food resources and improved acquisition techniques specifically tailored to local environments, together with the onset of intentional cultivation of certain plants, become parts of viable food acquisition strategies, as is well-documented in southwestern Asia^1,10,15^. North China data, as well, indicate that similar measures for broadening the subsistence spectrum were adopted, as were new technologies such as microblades and grinding stones, and eventually pottery.

A prerequisite for domestication is the availability of suitable plant resources in a given area. Analysis of surface soil δ ^13^C_TOC_ indicates that the area between latitudes 31°N to 40°N in the eastern North China Plain, where dry-land farming evolved, was originally covered by C3/C4 metabolic pathway mixed vegetation communities^42^. Although we cannot identify the species represented by the grass starches, it is known that Triticeae include early-maturing C3 grasses that poorly sustain frequent droughts^43^. As early maturing plants, humans could not rely on them for winter survival without using storage facilities, which are unknown for the Late Pleistocene. In contrast, grasses of the Paniceae tribe are warm-season and more drought-resistant C4 plants that mature in the fall and have a wider seasonal window for their growth, and thus could more successfully sustain climatic fluctuations during the Holocene^28,44,45^. Their ecological attributes make them available for human consumption during a longer period during the year, especially in the summer and fall seasons. Under any conditions, increasing reliance on cereals required the development of storage. The 8000–7500 cal BP Cishan deep storage pits demonstrate that this technique for conserving grain for winter season consumption and for security against bad seasons was already in place^23,26^. One can predict that older installations will be found in more Terminal Pleistocene sites such as Donghulin and Nanzhuangtou as archaeologists become more aware that such features can be present.

**Figure 3:**
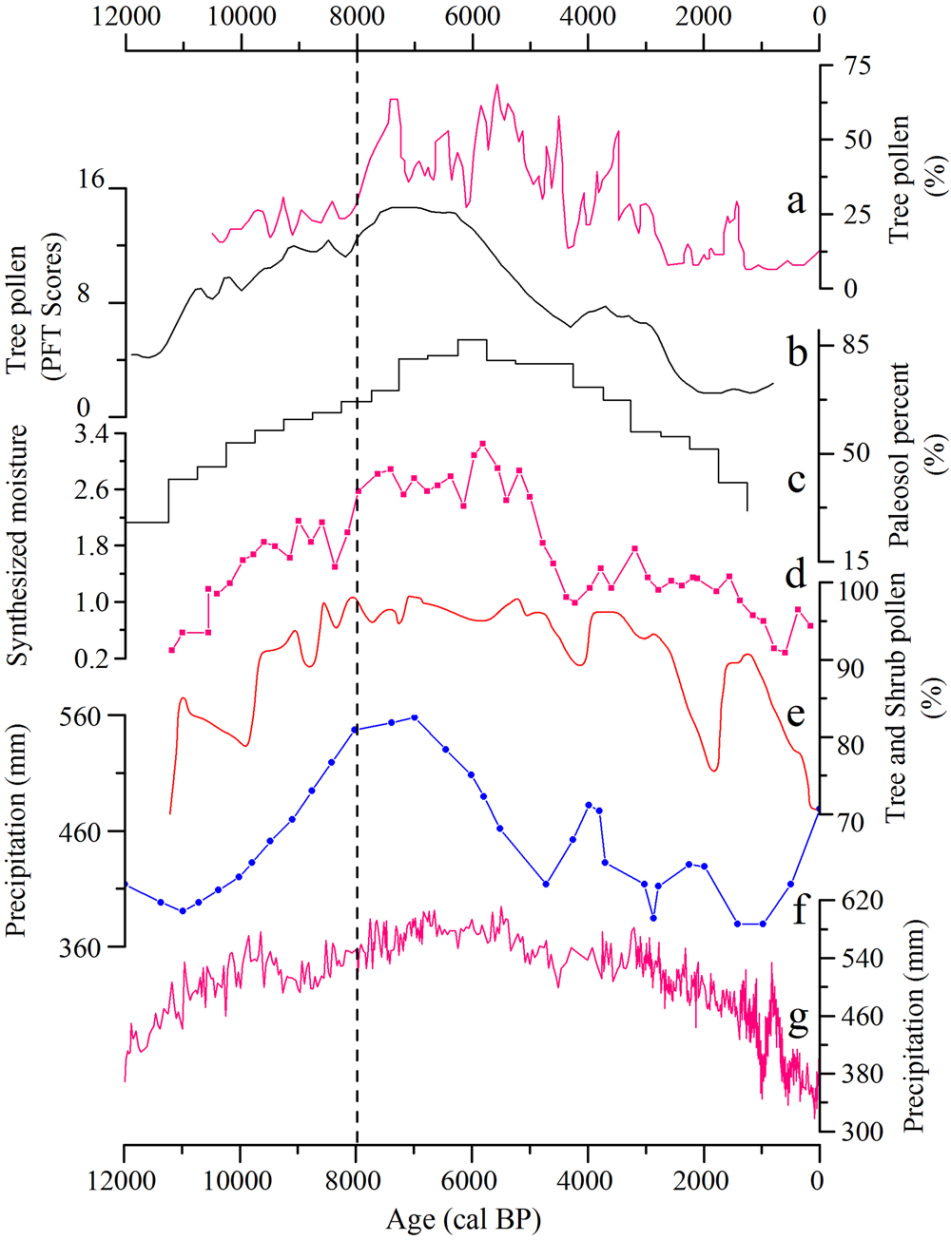
Paleoclimate reconstructions from different locations and proxies in northern China. (**a**) Tree pollen percentages from Daihai Lake^50^; (**b**) Tree pollen percentages from Bayanchagan Lake^51^; (**c**) Reconstructed moisture evolution based on paleosol development in the deserts and Sandy Lands of northern China^52^; (**d**) A moisture record based on the percentages of trees and shrubs in the semi-arid belt of northern China^53^; (**e**) Tree and shrub percentages from Erlongwan Maar Lake^54^; (**f**) Quantitative precipitation reconstruction from Lake Luanhaizi^55^; (**g**) Pollen-based annual precipitation reconstructed from Gonghai Lake^56^. The dashed line indicates the onset of the Holocene Climate Optimum around 8000 cal BP.

With the onset of Holocene conditions, global temperatures increased^46–48^ (Fig. 2c–e). The rise of both temperature^49^ (Fig. 2f) and precipitation in North China^50–56^ (Fig. 3a–g) were the result of an increase in the intensity of the East Asia monsoon cycle driven by mounting summer solar insolation (Fig. 2g)^57^ and rising sea level^52^. The augmented monsoon precipitation concentrated mainly during the warmer seasons, thus corresponding to the growing season of the millets^58^. Under the generally favorable climatic conditions during the early Holocene, the availability, abundance, and predictability of grasses of the Paniceae tribe improved. Records of δ^13^C from several loess/paleosol sections on the eastern and central Loess Plateau indicate an apparent increase of up to 40% in the abundance of C4 plants relative to C3 plants from the Late Pleistocene to the Holocene^59,60^. The amplified growth conditions and spatial distribution of the C4 plants of the Paniceae tribe caused their increased abundance and availability for foragers over the grasses of the Triticeae C3 species. When good conditions of preservation are encountered accidentally, such as in Shizitan 9^9^, the presence of seeds of wild *Setaria* and *Echinochloa* spp. demonstrates the botanical choices made by humans. These observations support the contentions of this paper. In addition, the rainfall patterns during the warm seasons assisted millet’s growth and increased its biomass productivity and therefore its annual yield potential. The combined natural effects would not escape the observations of local hunter-gatherers, who apparently preferentially selected the more profitable Paniceae grasses, leading to intentional cultivation and eventual domestication.

The earliest of the Late Pleistocene starch remains studied here are those from Longwangchan and Shizitan 14. During this first stage, our data indicate that mobile foragers, with grinding stones and the new, specialized technology of microblades, took advantage of the differences in ecological and physiological traits of both grass groups to cope with seasonal food shortages.

At some point, as collection of grasses for food intensified, cultivation begins. While it is argued that cultivation in southwestern Asia may have started during the Younger Dryas^1,15^, Ohalo II shows there were plants initially cultivated 10,000 years earlier^14^, but perhaps this initial practice was abandoned and a second cultivation event occurs again with worsening environment during the Younger Dryas. A Younger Dryas mechanism was perhaps active in China, as well, as Paniceae starch evidence from Donghulin likely indicates that foxtail millet and/or its wild progenitor are probably undergoing cultivation^5^. This hypothesis is also supported by carbonized foxtail millet seeds at the site, described as demonstrating an early stage of domesticated morphology^61,62^. These observations point to the cold and dry conditions of the Younger Dryas, coupled with “relative population pressure” in a particular region, as the triggers for the start of small-scale cultivation of millets in North China.

During the dry but warming early Holocene (11,700–8000 cal BP), archaeobotanical evidence indicates continuous intensive utilization of seed resources from both tribes. Although only the millets were selected for domestication^5,7,61,62^, foragers in North China continued to exploit Triticeae grasses, but in decreasing amounts, as is documented by the archaeological evidence. This phenomenon may be related to climatic conditions during this period. Global paleoclimatic records demonstrate the enduring presence of large ice sheets in the Northern Hemisphere^52,56,63,64^. Climate-sensitive areas in North China^38,50,56^ (Fig. 3a–g) indicate that the early Holocene witnessed a moderate climatic amelioration including the rise of temperature and subdued precipitation when compared to the subsequent Holocene Climatic Optimum after c. 8000 cal BP. However, during the initial domestication of the millets around 9500–8500 cal BP by cultivators living in early permanently settled villages, the domesticated cereals’ seasonal yields were relatively low^5^. The continued but decreasing exploitation of Triticeae grasses needs to be considered within this context. Various scenarios for this type of co-existence are possible, including the continued presence of small, natural stands of Triticeae in favorable microenvironments near the sites or growing in plots primarily intended for millets that could be exploited, preference for longer-term storage of millets with consumption of Triticeae when it ripened in summer before the new millet crops matured, and the increasing entrenchment of cultural preferences for cereals and nutritional dependence on them.

As temperatures and precipitation increased with the Holocene Climatic Optimum after ~8000 cal BP, an intensified summer monsoon brought more rainfall to northern China during the warm seasons^58^. This climatic pattern provided favorable conditions that likely increased the competitive advantages of Paniceae grasses and resulted in more productive and reliable yields, likely coupled with increasing areas of more highly tended fields as millet systematic agriculture is adopted, thus reducing available stands of Triticeae. At the same time, planting, harvesting, storage, and processing technologies improved, as new cooking and other cultural practices evolved that favored millet cereal consumption, all perhaps driving human selection of millets with higher yields that could then produce more than the “normal surplus” in order to have domestic storage to sustain farming communities during seasons of scarcity^65^, as well as for newly emerging ritual demands. Cishan and related sites support this hypothesis by the evidence mentioned, such as the 5-meter deep storage pits containing an estimated 50,000 kg of grain crops, as well as the great numbers of grinding stones (which are also larger and more standardized in form than Late Pleistocene ones), more elaboration in pottery vessel types and greatly increased amounts of pottery, and more polished stone axes and adzes (for land clearance), and polished stone sickles^23,26,66^. Millets proved to be a more competitive cereal, with their fast growth and early maturity, along with their drought resistance and tolerance for poor soils, thus requiring fewer human inputs for cultivation^19^. At the same time, rising winter temperatures during the Holocene Climatic Optimum could have been unfavorable for the growth of Triticeae grasses adapted to a relatively cold winter or spring for their development.

In conclusion, we hypothesize that the interactions between the climatic fluctuations of the Terminal Pleistocene to early Holocene period and the particular physiological needs of grasses in both the Paniceae and Triticeae tribes resulted in their different relative availability, abundance, and yield stability after *ca*. 12,000 cal BP. These considerations very likely influenced the decisions of foragers, who then became cultivators, thus paving the road for millet domestication. The starch evidence presented here gives our first insights into cereal exploitation patterns from across the entire period of the lengthy domestication process, and these can serve to guide future research and the discovery of more macro-remains to support our hypothesis.

## Materials and Methods

### Lithic tools for examination

Ancient starches can be recovered from residues on the surfaces of tools, carbonized residues of potsherds, dental calculus, and cultural deposits. Here we selected just one type of lithic tool, the paired slab and muller (Fig. S4), as these grinding implements are used in food processing at sites in North China beginning in the Late Pleistocene and continuing through the Holocene. Their larger surface areas allow for higher chances of starch preservation than other, smaller tools. They also likely provide direct evidence of food consumption, assuming the processed cereal seeds were eaten. Limiting the study to these grinding tools allows us to look at the same food processing behaviors through time as they relate to the exploitation of starchy grasses. Archaeological experience and ethnography demonstrate that producing a bulk of cereal plant food to be made into porridge, steamed, baked, or brewed requires the use of grinding slabs or mortars.

### Sampling and Extraction of Starches

Tools from the sites of Longwangchan and Donghulin were sampled at the Key Laboratory of Land Surface Pattern and Simulation, Institute of Geographical Sciences and Natural Resources Research, Chinese Academy of Sciences. To allow for future studies of the same artifacts, one third of the length of each muller was sampled, as was one quarter of the slab.

To dislodge adhering sediment and starch, tools were shaken in an ultrasonic water bath for 5 to 10 minutes, and starch was then isolated using heavy liquid flotation with a solution of CsCl at a density of 1.8 g/cm^3^. The recovered residue was mounted in 10% glycerine and 90% water on a slide and examined with both white and cross-polarized light at a magnification of 400×. Starch grains were counted, analyzed for morphological features, then recorded and compared with those from the modern reference collection.

The lithic tools from the sites of Nanzhuangtou, Cishan, and Jiangjialiang were sampled at the storage rooms of the Heibei Provincial Institute of Archaeology and Cultural Relics in Shijiazhuang, Hebei, and the tools from the sites of Jian’gou, Luojiayingzi and Sanjianfang were sampled at the storage rooms of the Ongniud Banner Museum, Inner Mongolia. Tools selected for sampling were initially cleaned by brush to remove adhering dust from storage and then washed clean with ultra-pure water. Cavities on the surface of the tools were then targeted for residue removal. 20–40 microlitres of ultra-pure water were applied to the areas of interest and left there to hydrate for 3–5 minutes. The wetted area was agitated with a metal pin to dislodge the sediment within the cavities. Finally, a sample of this material was removed with a micropipette and transferred to a clean, new, snap-cap vial for storage. These samples were processed in the laboratory where starches were floated by CsCl according to the methods described above.

As a control for the presence of starch, during the previous studies, sediment samples were collected from the dust in the storeroom where the artifacts were curated, the surface soil at the sites, the sediments from the overlying layer and underlayer of the cultural deposits from which the artifacts were recovered, and the cultural deposits themselves. The samples were found to either lack starch granules completely or to have a density of starch that was much lower than the residues on the tools, indicating that the ancient starches were related to tool use^67^.

### Identification of ancient starches

We have compiled a modern reference collection of over 200 starch-producing species from 20 families that are common in China (http://cmsgd.igsnrr.ac.cn). All modern reference specimens were collected by the authors from botanical gardens and during field investigations. Starch grain identifications were based upon one-on-one comparisons between ancient starches and those derived from the modern reference collection.

Our starch keys and classifications emphasize attributes: overall grain shape; contour and surface features; position and form of the hilum and fissure, if any; number and characteristics of pressure facets; presence or absence of demonstrable lamellae; and mean length averaged from the measurement of 100–150 grains. Each starch grain observed under the microscope was photographed, then grouped based on morphological features.

Currently, the Tribe Triticeae includes 13 genera (one introduced) and 175 species (99 endemic, eight introduced) that have been identified in China; the Tribe Paniceae has 27 genera (one endemic, two introduced) and 145 species (16 endemic, 12 introduced) that occur in China (http://www.efloras.org/). The morphological features of seed starches from the tribe Paniceae are typically, but not always, polyhedral **(**Fig. S1**)**, and some species occasionally contained spherical grains. The hila of starch grains from the Paniceae are centric with occasional fissures. The sizes range from a few microns to more than 20 microns in maximum length.

Starch grains from seeds of the Triticeae occur in bimodal populations of small, non-diagnostic forms and large, diagnostic types that are lenticular in morphology, with a side view that is oval with both ends attenuated. The equatorial groove, a remnant of the formation process, is frequently visible in the side view, and small indentations occur on the surface of a portion of the large grains in a population (Fig. S1**)**. The mean size of starch grains from these tribes often fall into the range of 20–30 microns.

## Electronic supplementary material

Supplementary information
