# Supplementary material for: Critical role of climate change in plant selection and millet domestication in North China
Source: Sci Rep. 2018 May 18;8:7855. doi: 10.1038/s41598-018-26218-6 (PMC5959876; doi:10.1038/s41598-018-26218-6)
Supplement: Supplementary file 1 — Supplementary information [file 41598_2018_26218_MOESM1_ESM.doc]

**Critical role of climate change in plant selection and millet domestication in North China**

Xiaoyan Yang^1^, Wenxiang Wu^1*^, Linda Perry^2, 3^, Zhikun Ma^4^, Ofer Bar-Yosef^5^, David J. Cohen^6^, Hongbo Zheng^7^, and Quansheng Ge^1*^

^1^ Key Lab. of Land Surface Pattern and Simulation, Institute of Geographical Sciences and Natural Resources Research, Chinese Academy of Sciences, Beijing 100101, China

^2^ The Foundation for Archaeobotanical Research in Microfossils, Alexandria, VA, U.S.A.

^3^ Department of Anthropology, George Washington University, Washington, D.C., U.S.A.

^4^ School of Cultural Heritage, Northwest University, Xi’an 710069, China

^5^ Department of Anthropology, Harvard University, Cambridge 02138, MA, U.S.A.

^6^ Department of Anthropology, National Taiwan University, Taipei 10617, Taiwan

^7^ Research Center for Earth System Science, Yunnan University, Kunming 650091, China

*Correspondence to [wuwx@igsnrr.ac.cn](mailto:wuwx@igsnrr.ac.cn) or [geqs@igsnrr.ac.cn](mailto:geqs@igsnrr.ac.cn)

**Supplementary Information**

**Supplementary Figures 1-3**

**Supplementary Tables 1-7**

**Fig. S1** Modern and archaeological starch granules from the tribes Paniceae and Triticeae.

The upper row is modern starches from the genera *Setaria* (a-c), *Panicum* (d-e), *Coix* (f), *Echinochloa* (g) of tribe Paniceae. The middle row is modern starches from the *Hordeum distichon* var. *nudum* (h), *Triticum aestivum* (i), *Hordeum vulgare* var. *celeste* (j), *Hordeum vulgare* var. *trifurcatum* (k), *Hordeum agriocrithon* (l), *Aegilops tauschii* (m), those members of the tribe Triticeae and side view of the starch grains from the tribe (n). The lower row is typical archaeological starch grains identified as Paniceae (o-q) and Triticeae (r-u) from archaeological sites. Scale bar is 20 microns except marked.

**Fig. S2** Atmospheric circulation systems of China and the locations of paleoclimate records.

Black arrows indicate the wind directions of the East Asian winter monsoon, the Westerlies, the and the East Asian summer monsoon. The red dashed line marks the modern Asian summer monsoon limit. The paleoclimate records (black triangles) include (a) Luanhaizi Lake; (b) Gonghai Lake; (c) Daihai Lake; (d) Bayanchagan Lake; (e) Erlongwan Maar Lake.

**Fig. S3** Some grinding stone tools for study.

a, the earliest slab from the Longwangchan site, 25,000 yr BP b-c, slab and muller from the Donghulin site, 10,000-9,500 yr BP; d-e, slab and muller from the Cishan site around 8,000 yr BP. Scale bar is 5mm.

**Table S1** Starch grain frequencies and Percentages from the grinding slab excavated from Locality 1 of the Longwangchan Site (~25,000 yr BP)

**Table S2** Starch grain frequencies and Percentages from the grinding stone tools excavated from Locality 14 of the Shizitan Site (23,000-19,000 yr BP)

**Table S3** Starch grain frequencies and percentages from the grinding stone tools excavated from the Nanzhuangtou Site ( >11,000 yr BP)

**Table S4** Starch grain frequencies and percentages from the grinding stone tools excavated from the Donghulin Site (11,050-9,500 yr BP)

**Table S5** Starch grain frequencies and percentages from the grinding stone tools excavated from the Cishan Site (~8,000yr BP)

**Table S6** Starch grain frequencies and percentages from the grinding stone tools excavated from the Jiangjialiang Site (~7,700 yr BP)

**Table S7** Starch grain frequencies and percentages from the grinding stone tools excavated from the sites of Jian’gou, Luojiayingzi & Sanjiafang of the early-middle Hongshan Culture (6,500-5,500yr BP)



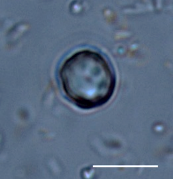

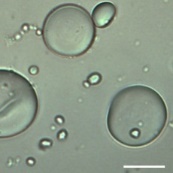

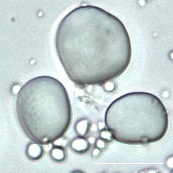

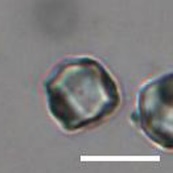

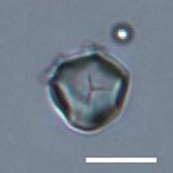

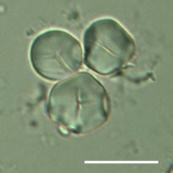

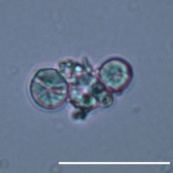

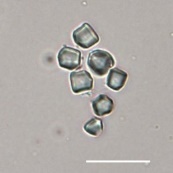

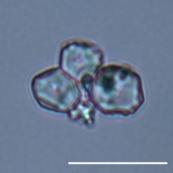

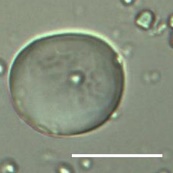

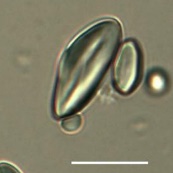

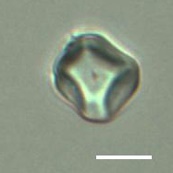

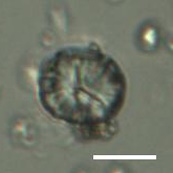

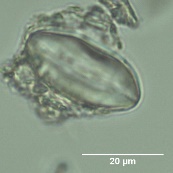

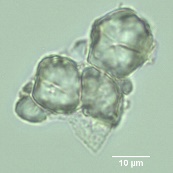

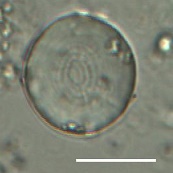

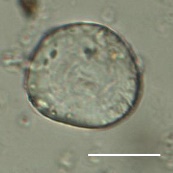

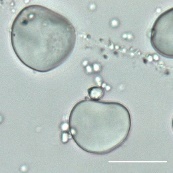

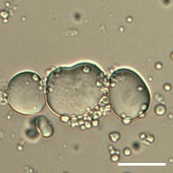

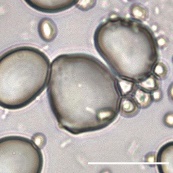

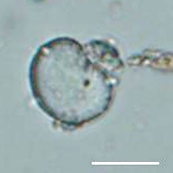


a

b

c

d

e

f

g

h

i

j

k

l

m

n

o

p

q

r

s

t

u

**Fig. S1** Modern and archaeological starch granules from the tribes Paniceae and Triticeae

**
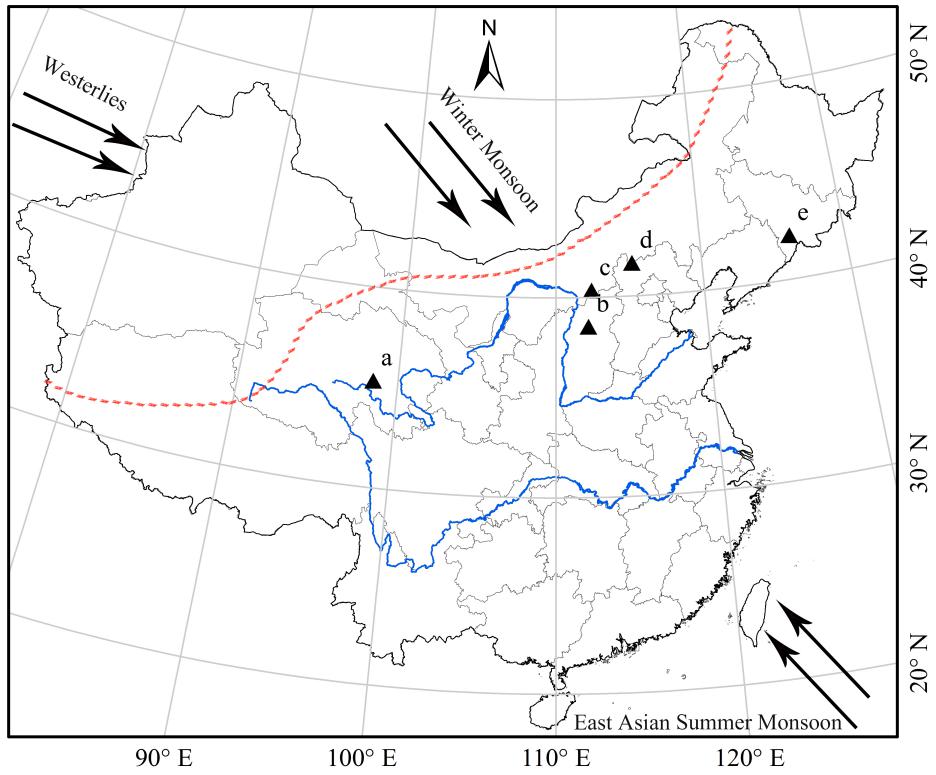
**

**Fig. S2** Atmospheric circulation systems of China and the locations of paleoclimate records. The figure S2 was generated using DIVA-GIS 7.5 (http://www.diva-gis.org/).

**Fig. S3** Some grinding stone tools for study.

**Table S1 Starch grain frequencies and percentages from the grinding slab excavated from Locality 1 of the Longwangchan Site (~25,000 yr BP)**

| **Samples** | **Field No.** | **Subsamples** | **Classification** | | | **Total** |  | | | |
| --- | --- | --- | --- | --- | --- | --- | --- | --- | --- | --- |
|  |  |  | **Paniceae** | **Triticeae** | **Others** | | |  |  | |
| Slab | 2005YHLWC I23T0208:1233 | U1 | 9 | 3 |  | | | 12 |  | |
|  |  | U2 | 3 |  |  | | | 3 |  | |
|  |  | U3 | 23 | 2 |  | | | 25 |  | |
|  |  | U4 | 5 | 1 |  | | | 6 |  | |
|  |  | N1 | 7 | 2 | 3 | | | 12 |  | |
|  |  | N2 | 5 |  | 1 | | | 6 |  | |
|  |  | N3 | 1 | 1 | 1 | | | 3 |  | |
| Total | |  | 46 | 9 | 5 | | | 60 |  |  |
| Percentage | |  | 76.7% | 15% | / | | | / |  |  |

**Table S2 Starch grain frequencies and percentages from the grinding stone tools excavated from Locality 14 of the Shizitan Site (23,000-19,000 yr BP)**

| **Samples** | **Field No.** | **Subsamples** | **Classification** | | | **Total** |  | | | |
| --- | --- | --- | --- | --- | --- | --- | --- | --- | --- | --- |
|  |  |  | **Paniceae** | **Triticeae** | **Others** | | |  |  | |
| Slab | GS1 | GS1-1 | 1 |  |  | | | 1 |  | |
|  |  | GS1-2 |  | 3 | 2 | | | 5 |  | |
|  | GS2 | GS2-2 |  | 3 |  | | | 4 |  | |
|  | GS3 | GS3-1 | 18 | 33 | 48 | | | 99 |  | |
|  |  | GS3-2 | 1 | 1 | 17 | | | 19 |  | |
|  |  | GS3-3 |  | 5 | 3 | | | 8 |  | |
| Total | |  | 20 | 45 | 71 | | | 136 |  |  |
| Percentage | |  | 15% | 33% | **/** | | | **/** |  |  |

**Table S3 Starch grain frequencies and percentages from the grinding stone tools excavated from the Nanzhuangtou Site ( >11,000 yr BP)**

| **Samples** | | **Field No.** | | **Subsamples** | **Millets** | **Triticeae** | | | **Others** | **Total** | |
| --- | --- | --- | --- | --- | --- | --- | --- | --- | --- | --- | --- |
| Slab | G3:110 | | used facet | | >109 | | 25 | 92 | | >226 | |
|  |  |  | unused facet | | 44 | | 8 | 26 | | 78 | |
| Muller | G3:367 | | used facet | | 32 | | 9 | 24 | | 65 | |
|  |  |  | unused facet | | 20 | | 10 | >9 | | >39 | |
| Total | | |  | | >205 | | 50 | >138 | | >408 |  |
| Percentage | | |  | | >50% | | 12.2% | / | | / |  |

**Table S4 Starch grain frequencies and percentages from the grinding stone tools excavated from the Donghulin Site (11,050-9,500 yr BP)**

| **Samples** | **Field No.** | | **Subsamples** | **Millets** | **Triticeae** | **Others** | **Total** | | | | | | |
| --- | --- | --- | --- | --- | --- | --- | --- | --- | --- | --- | --- | --- | --- |
| **Early Occupational phase** | | | | | | | | | | | |  |  |
| Muller 1 | T4(10):65 | | used facet | 76 | 28 | 6 | 110 | | | | | | |
|  |  |  | unused facet | 28 | 6 | 7 | 41 | | | | | | |
| Muller 2 | T8(8):2 | | facet A | 16 | 9 | 3 | 28 | | | | | | |
|  |  |  | facet B | 29 | 22 | 4 | 55 | | | | | | |
|  |  |  | facet C | 12 | 24 | 3 | 39 | | | | | | |
|  |  |  | facet D | 96 | 51 | 8 | 155 | | | | | | |
| Total | | | | 257 | 140 | 31 | | | | 428 |  | | |
| Percentage | | | | 60.0% | 32.7% | / | | | | / |  | | |
| **Late Occupational Phase** | | | | | | | | | | | |  |  |
| Muller 3 | T8(3):99 | used facet | | 3 | 4 | 3 | 10 | | | | | | |
|  |  | unused facet | |  |  | 1 | 1 | | | | | | |
| Pestle | TG2(4):2 | | | 6 | 10 | 4 | 20 | |  | | | | |
| Slab | T9(3):799 | | unused facet | 44 | 11 | 6 | 61 | | | | | | |
|  |  |  | used facet | 85 | 9 | 5 | 99 | | | | | | |
| Sherd | T9(5):1268 | | | 40 | 6 | 4 | 50 | |  | | | | |
| Total | | | | 178 | 40 | 23 | 241 |  | | | | | |
| Percentage | | | | 73.9% | 16.6% | / | / |  | | | | | |

**Table S5** Starch grain frequencies and percentages from the grinding stone tools excavated from the Cishan Site (~8,000yr BP)

| Samples | Field No. | Subsamples | Paniceae | Triticeae | Others | Total | |
| --- | --- | --- | --- | --- | --- | --- | --- |
| Muller | Group86WCT30②:8 | U-1 | 4 |  | 3 | 7 | |
|  |  | N-1 | 1 |  | 0 | 1 | |
| Slab | Group86WCT30②:12 | U-1 | 3 |  | 2 | 5 | |
|  |  | N-1 | 1 |  |  | 1 | |
| Muller | Group86WCT30③:40 | U-1 | 20 | 1 | 1 | 22 | |
|  |  | N-1 | 14 |  |  | 14 | |
|  |  | U-2 | 3 | 2 | 1 | 6 | |
| Muller | Grooup86WCT31⑥:2 | U-1 | 1 | 1 | 1 | 3 | |
|  |  | U-2 | 2 |  | 1 | 3 | |
| Slab | 87WCH49:7 | U-1 | 4 |  | 2 | 6 | |
|  |  | N-1 | 2 |  |  | 2 | |
|  | Group98WCT107:50 | U-2 | 4 |  | 3 | 7 | |
|  |  | U-3 | 3 |  |  | 3 | |
| Muller |  | U-1 | 4 |  | 2 | 6 | |
|  |  | U-2 | 1 |  |  | 1 | |
| Slab | Group86WCT31④:2 | U-1 | 3 |  |  | 3 | |
| Muller | 85WCH97:1 | U-1 | 6 |  |  | 6 | |
|  |  | U-1 | 1 |  | 1 | 2 | |
| Muller | 85WCH95:19 | U-1 | 8 |  | 3 | 11 | |
|  |  | U-2 | 10 |  | 2 | 12 | |
|  |  | U-3 | 11 | 1 | 2 | 14 | |
|  |  | U-4 | >5 | >4 |  | >9 | |
| Muller | Group98WCT107:22 | U-1 | 2 | 1 |  | 3 | |
|  |  | U-2 | 3 |  |  | 3 | |
| Total | |  | 116 | 10 | 23 | 149 |  |
| Percentage | |  | 77.9% | 6.7% | / | / |  |

**Table S6 Starch grain frequencies and percentages from the grinding stone tools excavated from the Jiangjialiang Site (~7,700 yr BP)**

| **Samples** | **Field No.** | **Subsamples** | **Paniceae** | **Triticeae** | **Others** | **Total** |
| --- | --- | --- | --- | --- | --- | --- |
| Slab | 95JJLF7:14 | U-1 | 11 | 1 |  | 12 |
|  |  | U-2 | 4 | 5 | 2 | 11 |
|  |  | U-3 | 6 |  |  | 6 |
|  |  | N-1 | 3 |  | 1 | 4 |
| Slab | 95JJLF7:44 | U-1 | 5 |  |  | 5 |
|  |  | U-2 | 1 | 1 | 2 | 4 |
|  |  | U-3 | 2 |  |  | 2 |
|  |  | N-1 | 1 |  |  | 1 |
|  |  | N-2 | 1 |  |  | 1 |
| Slab | 95JJLF5:7 | U-1 | 5 |  |  | 5 |
|  |  | U-2 | 6 |  |  | 6 |
|  |  | U-3 | 5 |  |  | 5 |
|  |  | U-4 | 8 |  | 1 | 9 |
|  |  | U-5 | 3 |  | 3 | 6 |
| Muller | 95JJLF5：16 | U-1 | 9 | 3 |  | 12 |
|  |  | U-2 | 5 | 1 |  | 6 |
|  |  | N-1 | 4 | 1 |  | 5 |
| Muller | 95JJLF5:16-1 | U-1 | 4 | 1 |  | 5 |
|  |  | U-2 | 1 |  | 1 | 2 |
|  |  | N-1 | 1 |  | 1 | 2 |
|  |  | U-3 | 19 |  |  | 19 |
|  | Total |  | 104 | 13 | 5 | 128 |
|  | Percentage |  | 81.3% | 10.2% | / | / |

**Table S7 Starch grain frequencies and pPercentages from the grinding stone tools excavated from the sites of Jian’gou, Luojiayingzi & Sanjiafang of the early-middle Hongshan Culture (6,500-5,500yr BP)**

| **Site** | **Samples** | **Field No.** | **Subsamples** | **Paniceae** | **Triticeae** | **Others** | **Total** | |
| --- | --- | --- | --- | --- | --- | --- | --- | --- |
| Jian’gou | Muller | 150426-0326-5002 | U | 49 | 2 | 1 | 52 | |
|  |  |  | N | 10 |  |  | 10 | |
|  | Muller | 150426-0328-5002 | U | 51 |  | 1 | 52 | |
|  |  |  | N | 12 | 1 |  | 13 | |
| Luojiayingzi | Muller | - | U | 34 |  | 1 | 35 | |
|  |  |  | N | 26 |  |  | 26 | |
|  | Muller | - | U | 32 |  | 1 | 33 | |
|  |  |  | N | 25 |  |  | 25 | |
| Sanjianfang | Muller | 150426-0319-5001 | U | 27 |  |  | 27 | |
|  |  |  | N | 13 | 1 |  | 14 | |
|  | Muller | 150426-0321-5001 | U | 10 |  | 1 | 11 | |
|  |  |  | N | 1 |  |  | 1 | |
| Total | |  |  | 290 | 4 | 5 | 299 |  |
| Percentage | |  |  | 97.0% | 1.3% | / | / |  |
